# Evaluation and characterization of framycetin sulphate loaded hydrogel dressing for enhanced wound healing

**DOI:** 10.1371/journal.pone.0317273

**Published:** 2025-04-17

**Authors:** Zhuo Wu, Iqra Yaqoob, Mehreen Afzal, Furqan Muhammad Iqbal, Waseem Hassan, Xinjun Chen

**Affiliations:** 1 Department of Equipment Section, Shaanxi Provincial People’s Hospital, Shaanxi Province, Xian, China; 2 Department of Pharmaceutics, Bahauddin Zakariya University, Multan, Pakistan; 3 Department of Pathology, Nishtar Medical University, Multan, Pakistan; 4 Department of Pathology, Combined Military Hospital (CMH), Multan, Pakistan; 5 Department of Pharmacy, COMSATS University Islamabad, Lahore campus, Lahore, Pakistan; 6 Department of Emergency Medical Center, Xixian Campus of Shaanxi Provincial People’s Hospital, Shaanxi Province, Xian, China; COMSATS University Islamabad - Lahore Campus, PAKISTAN

## Abstract

**Background:**

Hydrogels loaded with antibiotics can be an effective drug delivery systemfor treating skin diseases or conditions such asinburns and wound healing.

**Objectives:**

The current research work was planned to preparea hydrogel dressing for an effective wound healing. The hydrogel formulation was aimed to provide sustained drug release, reducing the frequency of repeated applying the transdermal drug formulation or patch.

**Methods:**

Different polymers, polyvinyl alcohol, sodium alginate, and polyvinyl pyrrolidonein varying ratios were used to prepare hydrogels by freeze-thawing method. The prepared hydrogel formulations were loaded with framycetinsulphate (FC-S), a topical aminoglycoside.

**Results:**

Swelling behaviour, drug release pattern, wereinvestigated.Equilibrium and dynamic studies were conducted at *pH* 7.4. The prepared hydrogel formulations showed Euilibriumswellingratio of 197.5%. *The in-vitro* release pattern of FC-Shydrogels was determined by dissolution testing. The prepared hydrogels were characterized by scanning electron microscopy (SEM)andfourier transform infrared (FTIR)spectroscopy.Animal study was conducted on rats to evaluatethe *in-vivo* therapeutic effectiveness of FC-S hydrogels in wound healing. For that purpose,wounds were induced in the animals. The drug loaded hydrogel dressing was effiecent in wound heaing as the wound treated with FC-S loaded hydrogel was almost completely healed (97%) on the fifth day in comparison to commercially available product (Sofra Tulle gauze) that healed 86%, whereas free FC-S manifested healing at 76%.

**Conclusion:**

It was observed that hydrogel dressing loaded with FC-S was therapeutically more efficient and can be used as a potential candidate for wound healing.

## 1. Introduction

Transdermal drug delivery system (TDDS) is prepared to deliver effective therapeutic drug doses across the skin [1]. TDDS is preferable for drugs that are metabolized by the liver and decomposed in the stomach and intestine. The drug is directly absorbed into the systemic circulation avoiding first-pass metabolism eventually [2]. It avoids drug-drug, drug-food, and drug-gastrointestinal microflora interactions [3]. TDDS is suitable for old-agedpatients with limited oral activity [4]. It provides extensive benefits to the wound area. It is less traumatic to wounds and removes the excess exudates from wounds [5]. Thehydrogel dressing is a popular TDDS, due to its numerous advantages like higher entrapment effiecncy and sustained release characteristics [6].

Hydrogels are a hydrophilic, three-dimensional network of crosslinked polymers capable to swell and holdanlarge amount of water and biological fluids without losing their structure [7–11].The hydrogel can absorb wound exudates and provide a moist environmentthat is effective for wound healing. Hydrogel has semi-occlusive properties, it hydrate the wounds and has ability to rehydrate eschar, and helps in autolytic debridement [12]. Hydrogel dressings provide a convenient, non-invasive,cost-effective, and painless application with reduced dosing frequency and controlled release characteristics to provide prolonged delivery of the drug. They are more effeicient in comparison to other modern wound dressings, as they can be combined with mesenchymal stem cells for effective wond healing [13,14]. Different formulation methods of hydrogel preparation have been used by the pharmaceutical researchers, like ionic gelation, radiations and freeze-thawing [15–17]. Microwave radiations have been used successfully for hydrogel synthesis [18]. Hydrogels prepared by the freeze-thawing method have great potential for biomedical uses [19,20]. A literature survey revealed that various skin infections are treated with neomycin Sulphate (S) and gentamycin S-loaded hydrogel patches [21].

In this current research work,FC-S-loaded hydrogel dressing was prepared and evaluated for wound healing effect. FC is a broad-spectrum bactericidal antibiotic belonging to the class of aminoglycoside.Its Chemical Formula

**Fig 1:**
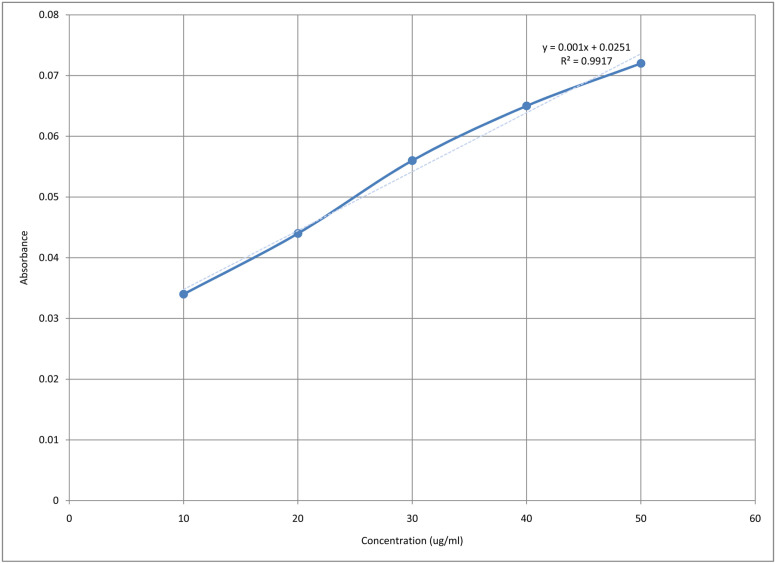
Calibration curve of Framycetin Sulphate.

C_23_H_46_N_6_O_13_ and structure is given as Fig 1. FC class III drug, BCS that is white to off white powder with melting point 160 °C, freely soluble in water, very slightly soluble in alcohol and practically insoluble in acetone.Themechanism of the action of inhibition includes the interruption in peptide-chain elongation, which blocks the A site of ribosomes leading to the genetic code misreading. FC-S has an effeicientwoundhealingand could be used as an alternative to silversuphadiazine, as it providespainless application without causingnephrotoxicity, ototoxicity and skin discoloration [22]. FC-Sis availableas cream, gel and ointment. It is also commercially available as guaze dressing for wound healing. It has been also studied by researchers as microsponge loaded gel for its efficient wound healing effect [23]. However, many studies have been conducted on preparing hydrogel dressings for wound healing, we selected a conveinient and economical method for preparing FC-S hydrogels without using crosslinker. This present study was aimed to formulate a TDDS of the FC-S-loaded hydrogel dressing by freeze thawing method. The FC-S hydrogel dressing was designed to provide sustained drug release, reducing the frequency of application that maintains the drug stability in polymeric network. FC-S-loaded hydrogel dressing composed of drug/polyvinyl alcohol/Polyvinyl pyrrolidone/sodium alginate by the freeze-thawing method has an advantage of physical crosslinking without using crosslinker. It avoids the possible toxic effect due to the crosslinking agent used in the preparation of chemically crosslinked hydrogels [24,25]. The prepared with FC-Shydrogel dressings were evaluated by different characterization techiques including FTIR, SEM, swelling behaviour, *invitro* drug release and *invivo* wound healing studies. The wound healing characteristics were studied in comparison to a commercial product, Sofra Tulle gauze.

## 2. Materials and methods

### 2.1. Materials

Polyvinyl alcohol (PVA) (average Mw=85000–124000, 99+%hydrolyzed), Polyvinyl alcohol (Average Mw 72000) from Sigma Aldrich, polyvinyl pyrrolidone (PVP) having Average Mw 40,000), were bought from Sigma-Aldrich. Sodium alginate, monobasic potassium phosphate, and sodium hydroxide werepurchased from UNI-CHEM.Allchemicalsusedwere of analytical grade.

### 2.2. Synthesis of FC-S-loaded hydrogel dressing

Various concentrationsofpolyvinylalcohol, polyvinyl pyrrolidone, and sodium alginatewereused and different formulations of hydrogel dressingswereprepared. The composition of hydrogel is shown in Table 1. The various formulations of hydrogel wound dressings were prepared by dissolving a accurately weighed 1 g as mention in Table 1. of polyvinyl alcohol by continuously stirring at 100–150 rpm at 80°C temperature for 1 hour as described [26]. Predetermined sodium alginate amount weighed and dissolved in distilled water by continuous stirring at 60°C for 15 minutes at 80rpm. Polyvinyl pyrrolidone was weighed and dissolved in distilled water. Polyvinyl alcohol (PVA) solution was mixed in already prepared sodium alginate solution (SA) and polyvinyl pyrrolidone solution and again stirred for 1 hour. The resulting solution was placed in a sonicator to remove the air bubbles. A weighted amount of FC-Swasadded to it. The resulting solution was poured intopetri dishes. These petri disheswereplaced for freeze-thawing and frozen at -20°C for twenty hours and then at room temperature. Hydrogels were thawed for three consecutive cycles of freeze-thawing. After thawing these prepared hydrogels were washed with distilled water to remove the unreacted polymer. And then hydrogelswereplaced on blotting paper to remove the excess water. FC-S-loaded hydrogels were prepared with both PVA (average Mw=85000–124000, 99+ % hydrolyzed) and were found more stable as compared to polyvinyl alcohol (Average Mw=72000) because of the higher molecular weight of PVA. It also remains stable at higher temperatures and is more strongly crosslinked with sodium alginate.

**Table 1:** Composition of FC-S-loaded hydrogel dressing.

Samples No	Quantity of Drug	Quantity of PVA	Quantity of PVP	Quantity of SA
**FH1**	0.1g	1.0g	0.7g	0.2g
**FH2**	0.1g	1.0g	0.6g	0.3g
**FH3**	0.1g	1.0g	0.5g	0.4g
**FH4**	0.1g	1.0g	0.4g	0.5g
**FH5**	0.1g	1.0g	0.1g	0.8g
**FH6**	0.1g	1.0g	0.2g	0.7g
**FH7**	0.1g	1.0g	0.4g	0.5g
**FH8**	0.1g	1.0g	0.5g	0.4g
**FH9**	0.1g	1.0g	0.6g	0.3g
**FH10**	0.1g	1.0g	0.7g	0.2g
**FH11**	0.1g	1.0g	0.8g	0.1g
**FH12**	0.1g	0.9g	0.9g	0.1g

#### 2.2.1. Measurement of drug entrapment efficiency.

DEE is typically determined by quantifying the amount of drug encapsulated within the hydrogel compared to the initial amount used. This is often achieved by dissolving a known quantity of the drug-loaded hydrogel in an appropriate solvent and measuring the drug concentration using analytical techniques such as UV-Vis spectroscopy or high-performance liquid chromatography (HPLC).

**Calculation:**


DEE (%)=(Initial amount of drug used Amount of drug encapsulated)×100

### 2.3. Characterization of FC-S-loaded hydrogels

#### 2.3.1. Fourier-transform infrared spectroscopy FTIR spectroscopy.

The hydrogel dried disk was crushed for FTIR by using pestle mortar.Powdered material of hydrogel in 1:100 proportion of potassium bromide was added mixed and then dried at 40 °C.Thin films of 1–2mm thickness wereobtained [27]. FTIRspectra were measured in the range of 3500–1000 cm^-1^. Spectra were recorded during twenty-four scans using f FT-IR BRUKER.

#### 2.3.2. *Scanning electron microscopy SEM.*

The morphological characteristics were determinedby using a scanning electron microscope (Evo LS 10 Zeiss Germany). Drug loaded hydrogels and drug free hydrogel formulutions were taken for SEM images to observe the changes illustrating the drug entrappedin hydrogel.

#### 2.3.3. Swelling ratio.

The swelling study was conducted by measuring the dynaminc swelling ratio and equilibrium swelling ratio to investigate the water absorption capacity of hydrogels dressings. Swelling behaviourcan be evaluated by using different buffering agents [28,29].

##### 2.3.3.1. Determination of dynamic swelling ratio (SR)

Hydrogel dressing made a network which has high and increased porosity. The ability of the hydrogel to imbibe an enormous quantity of water is shown as a swelling ratio of the hydrogel. The swelling ratio is an important requirement of hydrogel dressing for the dressing of wounds. Four formulations of the prepared hydrogel dressing PVA/PVP/SA loaded with FC-Swereselected for the swelling study. The selected (2 cm×2 cm) hydrogels were cut into pieces and dried at 45°C for 12 hours in a dry oven. Hydrogels were immersed in Phosphate-buffered saline (PBS) ofpH7.4. These swollen hydrogels were taken out from the solution at regular time intervals then placed on blotting paper and weighed the swollen gel and then these swollen hydrogels were put back in the same solution [30]. The swelling ratio of hydrogels was calculated by equation 1.


$${\text{SR}}_{\left(\text{Dynamic}\right)\text{=}}\left({\text{W}}_{\text{S}}-{\text{W}}_{\text{i}}{\text{/W}}_{\text{i}}\right)\text{×100}$$ (1)

Where the weight of swollen hydrogel is*W*_*s*_; *W*_*i*_ is the initial dried hydrogel weight [31–33].

##### 2.3.3.2. *Determination of Equilibrium swelling ratio SR*_*(Equilibrium)*_

Hydrogel dressing pieces were dried at 45°C for 12 hours. Then immersed these hydrogels in desired PBS of pH of 7.4 for 24 hours. These hydrogels in the swollen state from the solution were taken out and weighed until constant weight is achieved [34]. The equilibrium swelling ratio was calculated by using the formula written in equation 2.


$${\text{SR}}_{\left(\text{Equilibrium}\right)\text{=}}{\text{W}}_{\text{h}}{\text{/W}}_{\text{d}}$$ (2)

Percentage equilibrium swelling is determined by using the formula written in equation 3 [35,36]


$${\text{SR}}_{\left(\text{Equilibrium}\right)\text{\%=}}{\text{W}}_{\text{h}}{\text{/W}}_{\text{d × 100}}$$ (3)

Where ***W***_***h***_is the swollen hydrogel weight and ***W***_***d***_ is the weight of the hydrogel dried.

#### 2.3.4. *Calibration curve of FC-S.*

Stock solution 1 and stock solution 2 were prepared with a concentration of 1000µg/ml and 100µg/ml respectively. Different dilutions were prepared from stock solution 2, i.e., 10µg/ml, 20µg/ml, 30µg/ml, 40µg/ml, 50µg/ml by taking 1, 2, 3, 4, 5ml and make it upto 10 ml in volumetric flask. The solvent used was PBS, pH 7.4 By using UV spectrophotometer, these dilutions were analyzed at λ=258 nm [37]. Graphically shown in Fig 1.

#### 2.3.5. *In-vitro drug release studies.*

Four formulations of the synthesized hydrogel dressing polyvinyl alcohol/Polyvinyl pyrrolidone/sodium alginate loaded with FC-Swereselected for dissolution study on basis of greater water absorption characteristics measured byswellingratios.The selected formulations were FH5, FH9, FH11, FH12. The dissolution apparatus recommended by USP was used. Paddle apparatus was utilized to determine the release study. For this purpose,thehydrogelwasdried in an oven at 45°C for 24 hours. Dried hydrogels were immersed in100mL dissolution medium (PBS of pH of 4.4.4),ina dissolution apparatus beaker and the dissolution medium used. The dissolution medium was maintained at 32°C.4mL aliquots were obtainedat specified time intervals of 0.5, 1, 1.5, 2, 3, and 4 hours in dissolution medium is PBS 7.4 it is replenished with freshly prepared distilled water

also added throughout this procedure to keep the dissolution medium level up to the mark. Absorbance was analyzed by using an ultraviolet spectrophotometer for studying drug release patterns and compared with the standard curve of the drug used.For release kinetics of drug various models such as First order, Higuchi model, Zero order and Korsmeyer-Peppaswere used. Drug release mechanism studied in previous studies on hydrogels was fickian when n = 0.5, while the diffusion mechanism is called non-fickian when 0.5 <n<1 [16,38]

#### 2.3.6. *In-vivo wound healing test.*

*In-vivo* wound healing studies were carried on male wistarrats with the approval of Pharmacy Ethical Committee, Bahauddin Zakariya University Multan, Pakistan (No.187/PEC/2020) [39]. Twenty-fourmale healthy Wistarrats weighing 250–300 g were obtained from the animal house of the Faculty of Pharmacy, Bahauddin Zakariya University, Multan. The environment and cages were kept clean during the entire experiment. All the procedures were conducted in accordance to ARRIVE guidelines. Dorsal hairs of rats were shaved, the skin was cleaned with ethanol (70%), and anaesthetized with lignocaine gel. An abrasion wound (1.5 cm×1.5 cm) was induced by rubbing the sand-paper over the shaved skin and acetone was used untiloozing of skin wound and bleeding of skin starts. It was ensured that this procedure only damage the superficial part of the skin.

Rats were randomly divided into four groups of 6 rats each group. The wound of rats in Group A, Cand Dwas covered with FC-S-loaded hydrogel dressing having composition drug/polyvinyl alcohol/Polyvinyl pyrrolidone/sodium alginate (0.1/1/0.08/1),Sofra Tulle gauze, a sterile guaze which is a commercial product and drug-free hydrogel dressing having composition polyvinyl alcohol/Polyvinyl pyrrolidone/Sodium alginate (1/0.08/1), respectively. Group B was kept untreated (control). All the hydrogel dressings (2×2 cm) were applied on the wound area and fixed with an elastic adhesive bandage. All rats were kept in separate cages and dressing was changed every day for the experimental duration. Size of wound in cm was measured by measuring tape. The images of the wound were taken and preserved at 0, 1, 3, 4, and 5 day. Wound size reduction was calculated by using the equation 4.


$$\text{Reduction in wound size }\left(\text{\%}\right)\text{= }\left[\left({\text{W}}_{\text{Si}}{\text{-W}}_{\text{St}}\right)\right]\text{×100}$$ (4)

Where, ***W***_***Si***_ and***W***_***St***_ are the sizes of wound at initial time and ***t*** *time* respectively. Values were presented as mean ± SD (n-6). All the animals were released after complete wound healing.

## 3. Results and discussion

This study aims to synthesizebio-degradable and bio-compatible FC-S hydrogels. FC-S, a constituent of neomycin S [40] is an aminoglycoside with known pharmacological action in burn and woundtreatmet [22,41–43]. The visual aspect of hydrogel, FTIR, SEM, swelling studies, *in-vitro* drug release of hydrogel were performed for charaterization.Furthermore, *in-vivo* healing study was also performed on rats. As topical formulations of neomycin is already established in wound healing [44,45], the charaterisation of one of its constituent with advanced drug delivery system can be a useful.

### 3.1. Entrapment efficiency

The entrapment efficiency of optimized formulation is given in Table 2.

**Table 2:** Entrapment efficiency.

Sample Id	Initial drug	Encapsulated drug	Entrapment efficiency
**FH5**	**100mg**	**90**	**90%**
**FH9**	**100mg**	**87**	**87%**
**FH111**	**100mg**	**92**	**92%**
**FH12**	**100mg**	**95**	**95%**

#### 3.1.1. *FTIR.*

**Fig 2:**
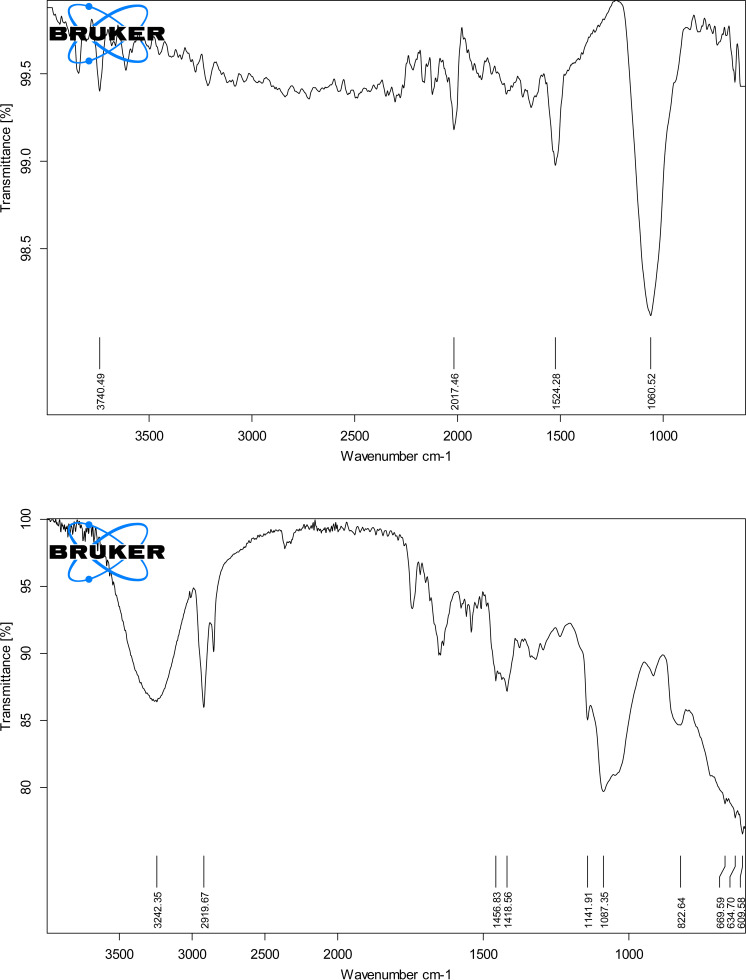
Fourier transform infrared (FTIR) spectra of framycetin sulphate (A) and drug- loaded hydrogel dressing (B).

FTIR spectra of FC-Sare shown in Fig 2(A). The peak at 3740 cm^-1^ isrelated to OH stretching and the peak at 2017 cm^-1^ shows N=C=S stretching. Similarly, peak at 1524 cm^-1^ represents N-O stretching while the peak at 1050 cm^-1^ relates to the stretching vibration of S=O.Thespectra of FC-S-loaded hydrogel dressingareshown in Fig 2 (B), where peaks at 3242 cm^-1^ is due to OH stretching vibrations and 2919 cm^-1^ due to C-H stretching. The peak at 1418 cm^-1^ depicts the O-H bending. At 1141 cm^-1^ S=O, indicates the stretching vibrations due to S group of FC-S, whereas, 1087 cm^-1^ absorbance frequency exhibit the C=O stretching due to secondary alcohol-related to PVA [18]. The FTIR spectra revealed successful cross-linking through significant shifts in characteristic wavenumbers. For instance, the –OH stretching vibration at ~3800 cm⁻¹ showed a shift to ~3740 cm⁻¹, indicating reduced free hydroxyl groups. Similarly, the C=O stretching peak shifted from ~1068 cm⁻¹ to ~1087 cm⁻¹, suggesting the formation of ester bonds. These shifts confirm the successful integration of cross-linking agents within the hydrogel network

#### 3.1.2. *Scanning Electron Microscopy:.*

**Fig 3:**
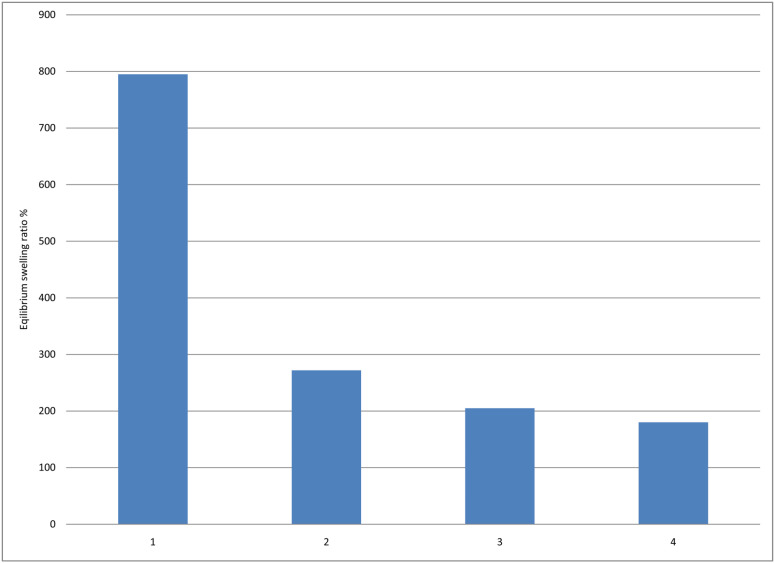
Equilibrium Swelling ratio of Polyvinyl alcohol/Polyvinyl pyrrolidone/Sodium alginate hydrogel dressing loaded with Framycetin Sulphate 1.FH5, 2.FH9, 3.FH11, 4.FH12.

In general, the SEM exhibits microstructure morphologies of hydrogels. The SEM of the synthesized hydrogel is shown in Fig 3. This picture verifies that the synthesized polyvinyl alcohol-based hydrogel in this work has a porous structure. In hydrogels existence of these pores strongly increases the swelling kinetics and drug release of the resulted product. The SEM images showed that the hydrogel was porous and pores were distributed uniformly. The porosity enables faster swelling which is useful for drug loading and release [11,18].

#### 3.1.3. *Swelling ratio:.*

**Fig 4:**
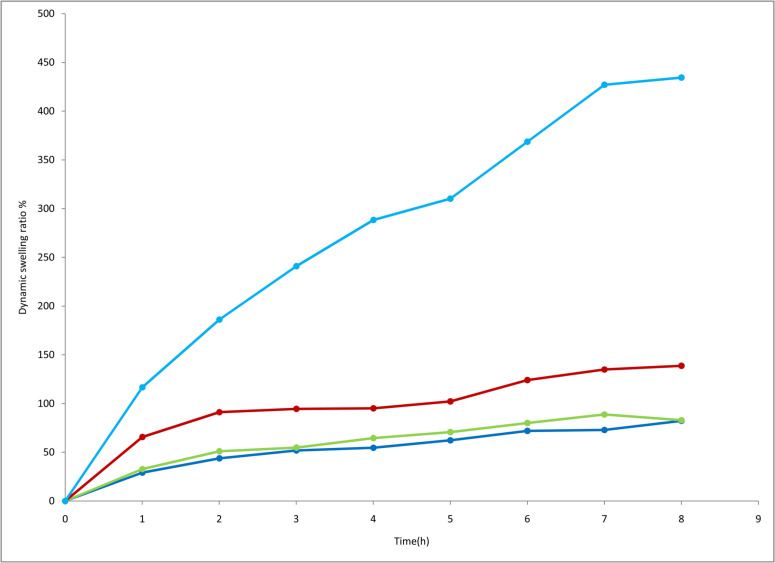
Dynamic swelling ratio (%) of Framycetin sulphate loaded hydrogel dressing of FH 5, FH9, FH11, FH12.

The swelling ratio of selected samples FH5, FH9, FH11and FH12 of FC-S-loaded hydrogels is shown in Fig 4 and Table 3. The hydrogels loaded with FC-S sample FH5 show maximum equilibrium swelling. Results indicate that sodium alginate showed greater swelling capacity than PVP. It suggests that the addition of sodium alginate increases the swelling capacity and has a positive effect on the equilibrium swelling ratio. FH5 Formulation has the highest weight ratio of sodium alginate which is1/0.1/0.8. By decreasing the sodium alginate ratio, swelling decreases [46].

**Table 3:** Percentage equilibrium swelling ratio SR data of FC-S loaded hydrogel dressing.

Time(h)	SR _(Equlibrium)%_	SR _(Equlibrium)%_	SR _(Equlibrium)%_	SR _(Equlibrium)%_
	FH5	FH9	FH11	FH12
24	758.69	259.25	211.95	180.01
48	793.47	277.77	217.39	197.5

**Fig 5:**
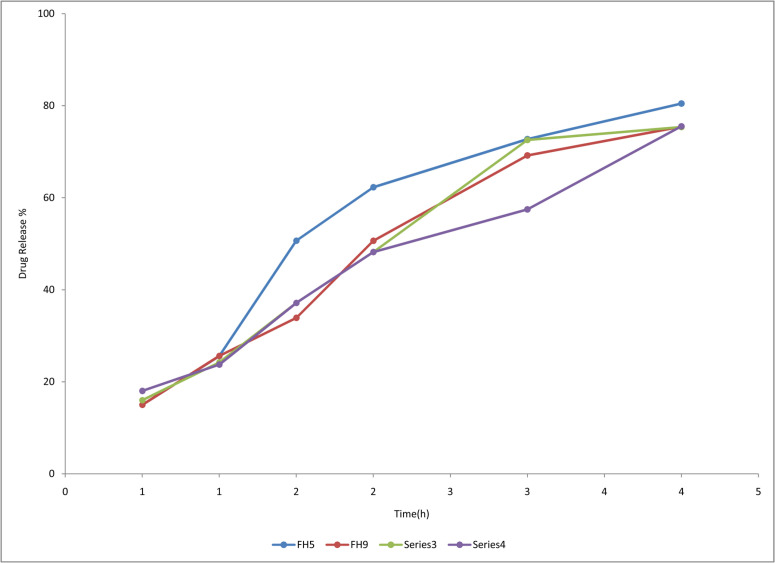
Drug release profile of FH5, FH9, FH11, FH12.

A dynamic swelling study was performed on four selected samples FH5, FH9, FH11, and FH12 having a ratio of Polyvinyl alcohol/Polyvinyl pyrrolidone/Sodium alginate(1/0.1/0.8), (1/0.6/0.3), (1/0.8/0.1), (0.9/0.9/0.1)with model drug FC-S 0.1g respectively. The most important hydrogel characteristic is swelling capacity without losing its shape and form from which water can not be removed under pressure [47]. The swelling ratio is affected by several factors such as hydrophilicity attributed to hydrophilic groups present on the polymer chain, polymer concentration and stiffness. These factors modify the average spaces in closslinked structure that affects the water absorption and structure. During the first duration of time in contact with solution rapidly swelling increases due to crosslinked polymer enabling to diffuse water quickly. The swelling ratio value of the hydrogels in this experiment was examined for 8 h at a time interval of 1h, and then at 24 h. It was observed that swelling ratio is affected by soaking time,the longer the period of soaking, the greater the swelling. It is according to the previously reported characteristics of hydrogels [48]. Dynamic swelling ratio observed for 8 hr, which is represented graphically and shown in Fig 5 and Table 4 for samples FH5, FH9, FH11, FH12. All the samples show a gradual increase in the swelling of hydrogel dressing. FH5 shows higher dynamic swelling because of sodium alginate due to more hydrophilic nature [49].

**Table 4:** Dynamic swelling ratio % of FC-S loaded hydrogel dressing.

Time(h)	SR (Dynamic)%	SR (Dynamic)%	SR (Dynamic)%	SR (Dynamic)%
	FH5 (PVA/PVP/SA)	FH9 (PVA/PVP/SA)	FH11(PVA/PVP/SA)	FH12(PVA/PVP/SA)
	**FH5**	**FH9**	**FH11**	**FH12**
1	118.19	62.96	23.01	22.5
2	187.82	86.29	44.02	42.5
3	238.47	87.03	49.45	47.5
4	293.49	88.14	60.32	50.6
5	313.04	100.1	65.21	57.4
6	376.08	118.1	73.36	67.5
7	441.3	129.62	80.55	70
8	443.47	137.03	82.06	75

#### 3.1.4. *In-vitro drug release studies.*

**Fig 6:**
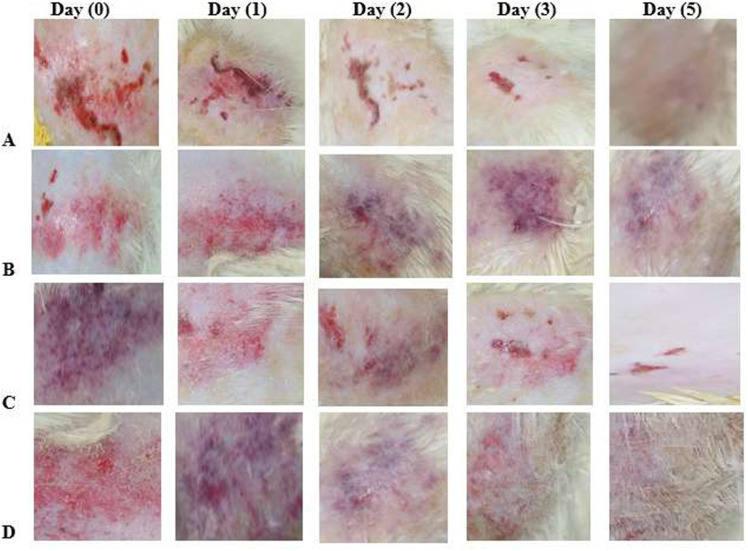
The images representing the wound on rat model (A) Treated with Framycetin Sulphate loaded hydrogel dressing (B) Non treated rat wound (C) Rat wound treated with commercial product Sufre Tulle gauze (D) Drug free hydrogel dressing treated rat wound. The framycetin sulphate loaded hydrogel dressing were composed of Framycetin sulphate drug polyvinyl alcohol, polyvinylpyrrolidone,sodiumalginate.

The *invitro* drug release of FC-S hydrogel dressings was determined, where, 50% of drug release was observed in all formulations in the 2 hours. Irrespective of polymers of dressing all the formulations were possessing the release rate of about 80% for 4 hours. The hydrogel dressing prepared with drug/PVA/PVP/SA of ratio 0.1/1/0.1/0.8, sample FH5 showed maximum drug release upto 88% in 4 hours. Other formulations FH 9, FH 11, and FH 12 showed drug release upto 80% after 4 hours as shown in Fig 6. It was showing the release of drug for longer period of time to provide a sustained effect. Drug release characteristics are related to water absoption capacity of hydrogels, ingress of aqueous mediumin hydrogel polymeric network eventually affects the release of drug [50]. To study drug release order, values of ʻʻR^2^ʼʼ were studied. The model for release of the drug is best when values of ʻʻR ^2^ʼʼare close toʻʻ1ʼʼ. In this release study, the values of ʻʻrʼʼ of most samples for release of the drug were close to ʻʻ1ʼʼ. So the model that best fits with FC-Srelease was first order. These values of (R^2^) and (K) have written in Table 4. However, values for ʻʻR^2^ʼʼ for higuchi model for drug release have exhibited a diffusion-controlled mechanism for the release of FC-S because when plotted against the fraction of drug release, the linear plot is obtained.

The slope and intercept of ʻʻ ln $\frac{Mt}{M\infty }$ ʼʼ in korsmeyerpeppas have been manipulated to determine the ʻʻnʼʼ value. The results for values of ʻʻnʼʼ have exhibited a non-fickian release of FC-S because values obtained were among 0.45–1. The drug release pattern of FC-S-loaded hydrogels FH5, FH9, FH11, and FH12 by employing different kinetics modelsisshown in Table 5. Table 6 exhibit the values of ʻʻnʼʼ. Drug release data for samples FH5, FH9, FH11, and FH12 are shown in Table 7.

**Table 5:** Drug release pattern of FC-S-loaded hydrogels FH 5, FH 9, FH11, FH 12 by employing different kinetics model.

Sample no	Drug/PVA/PVP/SA ratio (g)	Zero-order kinetics		First-order kinetics		Higuchi model	
		*Kot*	R^2^	*-K*_*1*_ *t*	R^2^	*K*_*2*_ *t* ^*1/2*^	R^2^
**FH5**	0.1/1/0.1/0.8	25.843	0.8242	0.478	0.9141	42.508	0.8195
**FH9**	0.1/1/0.6/0.3	22.339	0.9388	0.362	0.9618	36.402	0.8508
**FH11**	0.1/1/0.8/0.1	22.843	0.9278	0.376	0.9571	37.270	0.8499
**FH12**	0.1/0.9/0.9/0.1	21.015	0.9257	0.330	0.9646	34.412	0.8747

**Table 6:** Release exponent (n) and order of release of FC-S-loaded hydrogel dressing.

Sample no	Drug/PVA/PVP/SA ratio (g)	Release exponent (n)	R^2^	Order of release
FH5	0.1/1/0.1/0.8	0.735	0.8946	Non-fickian
FH9	0.1/1/0.6/0.3	0.814	0.9691	Non-fickian
FH11	0.1/1/0.8/0.1	0.803	0.9626	Non-fickian
FH12	0.1/0.9/0.9/0.1	0.775	0.9738	Non-fickian

**Table 7:** Drug release data of FC-S loaded hydrogel-dressing sample FH 5, FH 9, FH 11, FH 12.

Time (h)	Drug release (%) of FH5	Drug release (%) of FH9	Drug release (%) of FH11	Drug release (%) of FH12
0.5	9.41	9.71	11.31	14.61
1	24.81	27.71	26.1	23.11
1.5	55.99	35.21	40.1	40.11
2	69.61	54.22	50.71	50.21
3	77.66	70.45	76.61	57.6
4	88.55	80.21	79.81	79.8

#### 3.1.5. *In-vivo* wound healing on rats.

**Fig 7:**
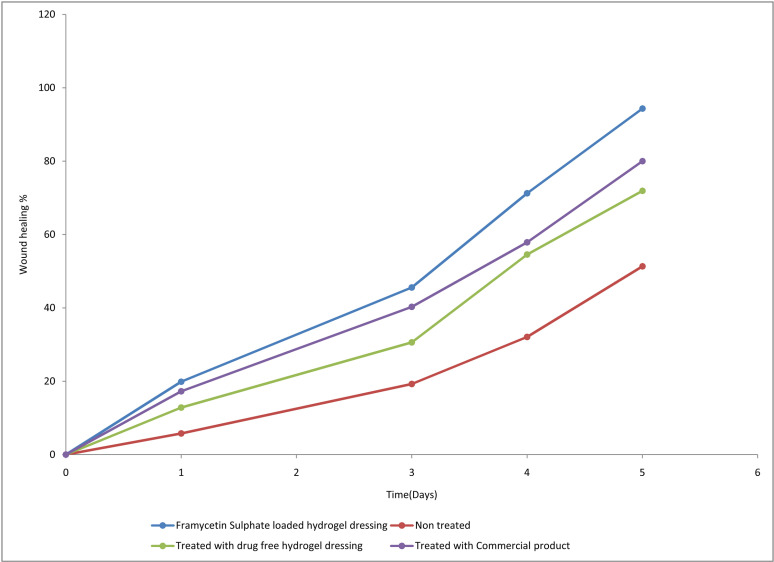
Wound curing profile of rats (wound model) Treated with framycetin sulphate loaded hydrogel dressing treated rat, Non treated rat wound, rat wound treated with commercial product Suffre Tulle gauze, Drug free hydrogel dressing treated rat wound. The framycetin sulphate loaded hydrogel dressing were composed of framycetin sulphate drug/Polyvinyl alcohol/Polyvinyl pyrrolidone/Sodium alginate ratio.

**Fig 8:**
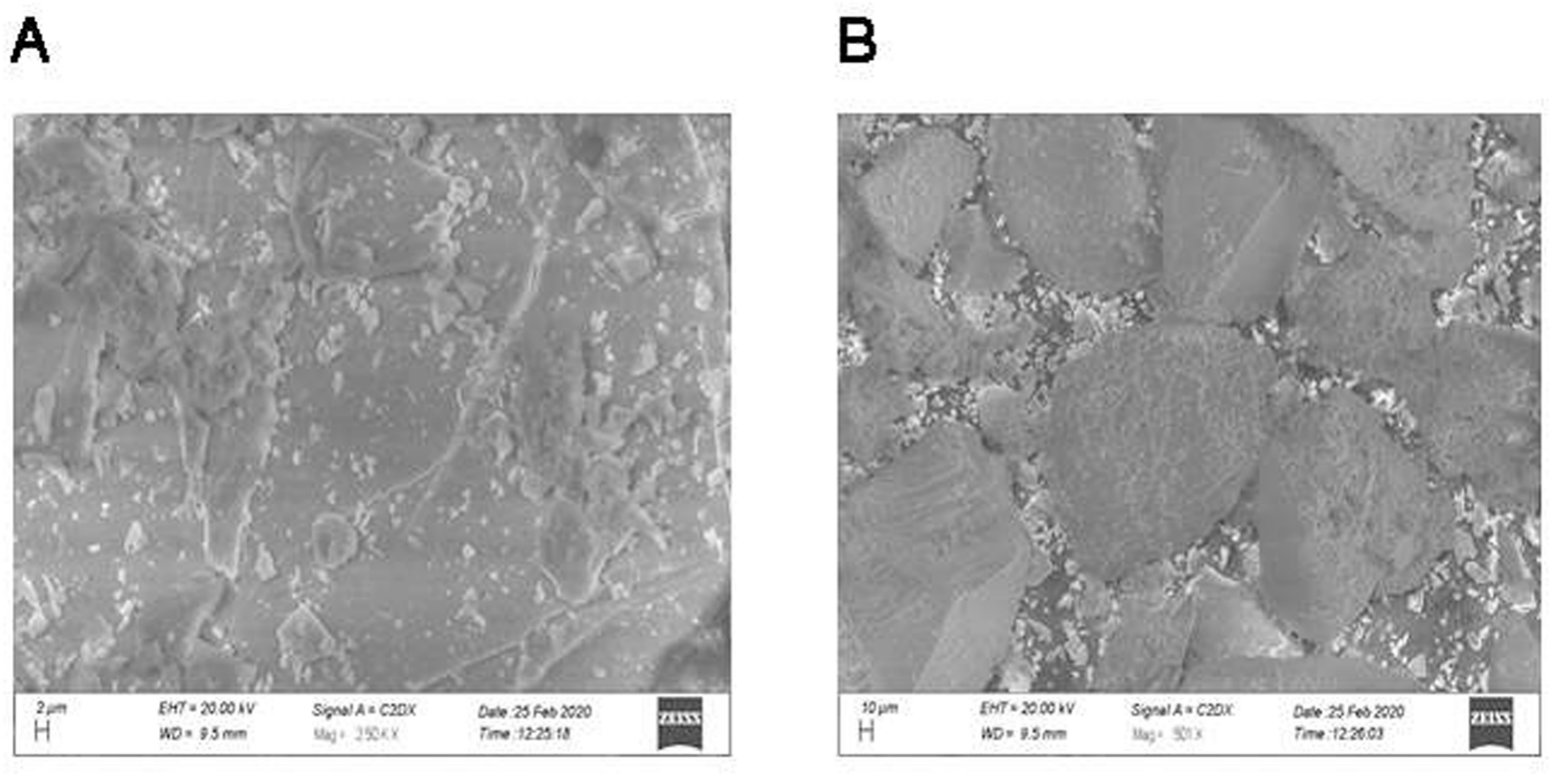
Scanning electron microscopic images of unloaded hydrogels (A) and loaded (B) dressingsdressings.

The natural mechanism of regenerating epidermal and dermal tissues is called wound healing. The mechanismsof tissue healing have been characterized into phases which involve the proliferative, inflammatory phase, and remodelling phases. In the inflammatory phase, debris and bacteria are removed and phagocytosis, mediator and cytokines are released that cause division of cells and migration including in the proliferative phase. Collagen, angiogenesis, granulation, deposition, epithelization, tissue formation and wound contraction occurs in the proliferative phase. To cover the wound, the epithelial cells start moving across during epithelization. By the process of wound contracture, the wound is eventually closed by a combination of all these. Collagen is realigned and remoulded in the maturation and remodelling phase, along tension lines and cells removed by the method of apoptosis. The images representing the wound on the rat modelareshown in Fig 7 and the wound curing profile is shown in Fig 8. Wound treated with drug-free hydrogel dressing (D), treated with hydrogel dressing loaded with FC-S (A) and treated with commercial product showed higher healing rate (C) and exhibited accelerated wound curing as compared to non-treated rat wound (B). Wound size reduction is given in Table 8 and percentage wound healing is numbered in Table 9. The commercial product sufre tulle treated rat wound showed accelerated curing and high curing rate compared to non-treated wound and treated with drug-free hydrogel dressing [51]. From the initial point at day 2–5 wound healing rate was higher in FC-S-loaded hydrogel as compared to the non-treated wound on rats, treated with a commercial product, and wounds treated with drug-free hydrogel dressing [46]. On days 3 and 5 wound healing rate was highest by FC-S-loaded hydrogels. At day 5 healing of wound rate in non-treated rats, wounds treated with drug-free hydrogel dressing, wounds treated with FC-S loaded hydrogel and wounds treated with commercial product were 50, 70, 86, 78% respectively. Results exhibited that wound treated with FC-S-loaded hydrogel shows maximum wound healing. Such excellent wound healing effect by FC-S-loaded hydrogel was mainly due to a moist environment with good swelling capacity. A moist environment was very helpful for the migration of keratinocytes, cell growth factors, and cytokine fibroblast [52]. Moreover,theFC-S model drug used is an aminoglycoside antibiotic which has a positive effect on wound healing because of its established mechanism of action on bacteria. Sodium alginate in hydrogel maintainsamoist environment that helps to increase healing and the making of granulation tissue. TheFC-S loadedhydrogel dressing was evaluated as efficient TDDS in curing wounds in comparison to the commercially available sterile guaze due to its firm adhesionand mechanical protection. The polymeric network due to the shape adaptability characteristics on contact with skin, that provide a sufficient coverage needed for safeguarding of wound [53,54].

**Table 8:** Wound size reduction in rat treated with FC-S hydrogels, nontreated, commercial product and drug free hydrogels.

Time (days)	0	1	2	3	5
**A.FC-S loaded hydrogel dressing size (cm)**	1.5	1.2	0.8	0.4	0.0
**B.Non treated (cm)**	1.5	1.4	1.2	1.0	0.7
**C.Commercial product (cm)**	1.5	1.25	0.9	0.6	0.2
**D.Hydrogel dressing without 0 drug**	1.5	1.3	1.05	0.65	0.35

**Table 9:** Percentage wound healing on rats model ingroups A, B, C and D.

Time (Days)	Treated with FC-S hydrogel dressing	Non treated	Treated with suffre tulle (commercial product)	Treated with drug free hydrogel dressing
	(A)	(B)	(C)	(D)
0	0	0	0	0
2	20	6	17	13
3	46	20	40	30
4	73	33	60	56
5	97	53	86	76

## 4. Conclusion

In current research, FC-S hydrogel dressinghave been developed by a physical cross-linking of PVA, PVP, and SA by freeze-thawing method. It can be concluded that preparedFC-S incorporated hydrogelshad high porosity and desriable morphological characteristics, suitablely possessing high swelling capacity in aqueous solution at pH 7.4. The swelling of a polymeric network was not markedly affected by composition of polymers,howeverSA has a minor effect on swelling behaviour. The hydrogel dressing had high ability to hold drug and able provide a sustained release characteristics. FC-S-loaded hydrogel dressing displayedimproved wound healing results than a commercial product due toimprovedmoistenvironment and wound exudates absorbing capacity. Furthermore, this hydrogel dressing would be expected to have better patient compliance as the application of local preparation would substantially decrease side effects systemically.

## Supporting information

S1 File FH 5 swelling.
(XLSX)

S2 File FH 11 swelling.
(XLSX)

S3 File FH9 swelling.
(XLSX)

S4 File FH12 swelng.
(XLSX)

S5 File FH5 drug release.
(XLSX)

S6 File FH9 drug release.
(XLSX)

S7 File FH11 drug release.
(XLSX)

S8 File FH12 drug release.
(XLSX)

S1 Data FTIR_data.
(DOCX)
