# Supplementary material for: Evaluation and characterization of framycetin sulphate loaded hydrogel dressing for enhanced wound healing
Source: PLoS One. 2025 Apr 17;20(4):e0317273. doi: 10.1371/journal.pone.0317273 (PMC12005552; doi:10.1371/journal.pone.0317273)
Supplement: S1 Data — FTIR_data. (DOCX) [file pone.0317273.s009.docx]

Drug framycetin sulphate

Drug loaded framycetin sulphate hydrogel dressing
